# Correction: Genomic variations of the mevalonate pathway in porokeratosis

**DOI:** 10.7554/eLife.14383

**Published:** 2016-01-27

**Authors:** Zhenghua Zhang, Caihua Li, Fei Wu, Ruixiao Ma, Jing Luan, Feng Yang, Weida Liu, Li Wang, Shoumin Zhang, Yan Liu, Jun Gu, Wenlian Hua, Min Fan, Hua Peng, Xuemei Meng, Ningjing Song, Xinling Bi, Chaoying Gu, Zhen Zhang, Qiong Huang, Lianjun Chen, Leihong Xiang, Jinhua Xu, Zhizhong Zheng, Zhengwen Jiang

Zhang Z, Li C, Wu F, Ma R, Luan J, Yang F, Liu W, Wang L, Zhang S, Liu Y, Gu J, Hua W, Fan M, Peng H, Meng X, Song N, Bi X, Gu C, Zhang Z, Huang Q, Chen L, Xiang L, Xu J, Zheng Z, Jiang Z. 2015. Genomic variations of the mevalonate pathway in porokeratosis. e*Life* **4**:e06322. 10.7554/eLife.06322.

Published 23 July 2015

In **Figure 3**, “*FDPS* (NM_001135822.1): c.468+1G>A” should be “*FDPS*: c.486+1G>A”.

The corrected **Figure 3** is shown here:

**Figure:**
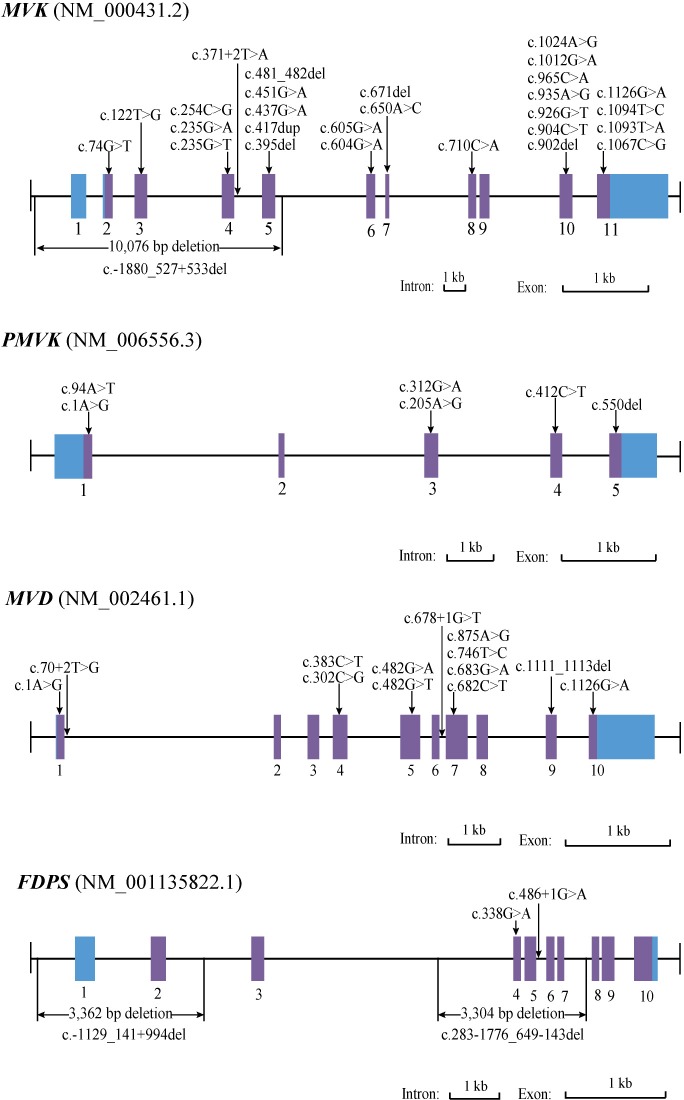

The originally published **Figure 3** is also shown for reference:

**Figure:**
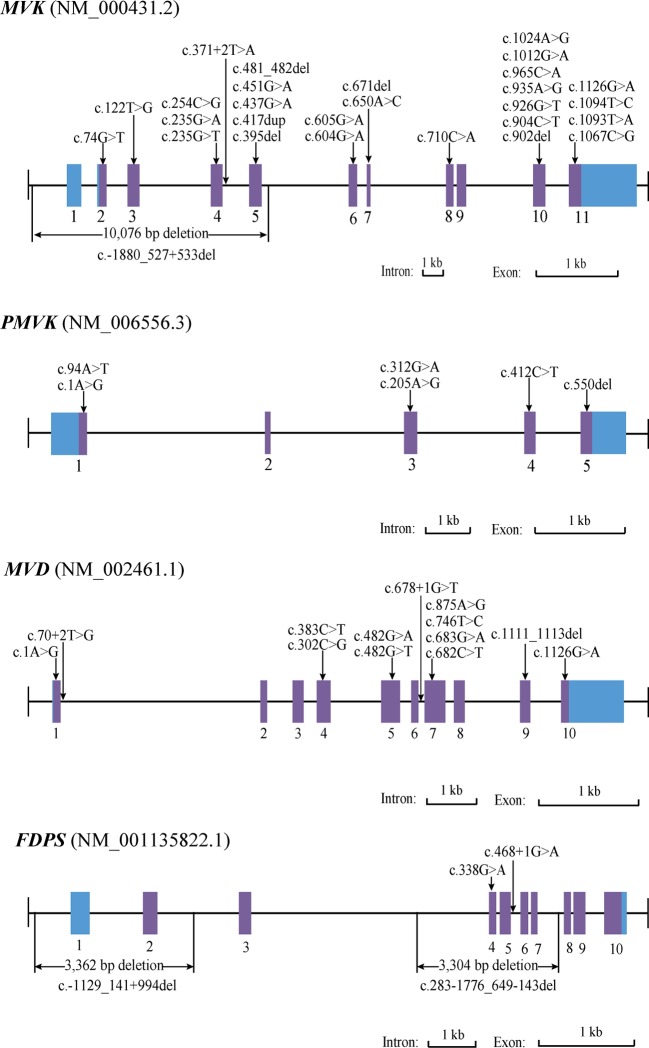

In **Figure 7**, “S-36 (*FDPS*: c.684+1G>A)” should read “S-36 *FDPS*: c.486+1G>A”.

The corrected **Figure 7** is shown here:

**Figure:**
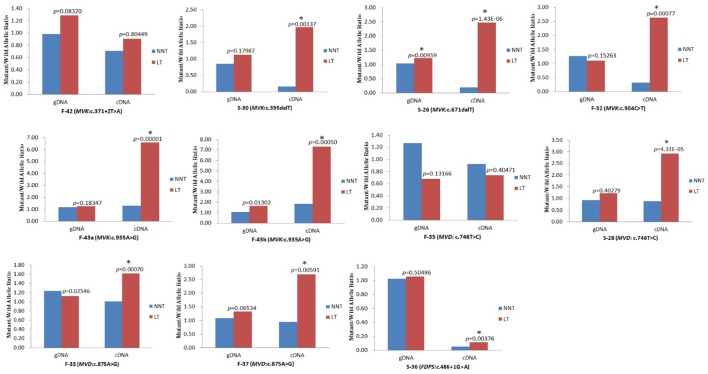

The originally published **Figure 7** is also shown for reference:

**Figure:**
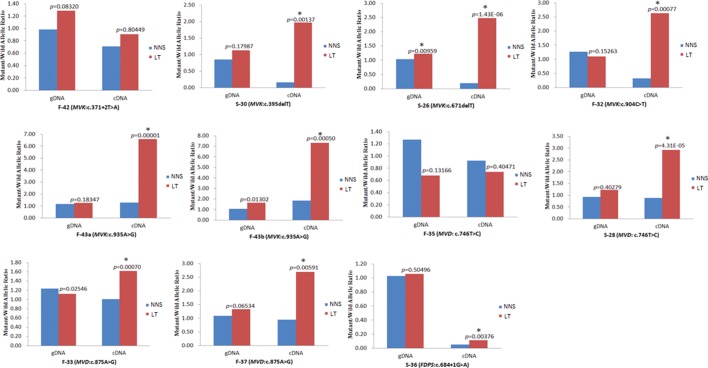

In addition, in **Supplementary File 3**, patient number 12 with “*FDPS*: c.684+1G>A” should read “*FDPS*: c.486+1G>A”.

We checked the description of one splice site mutation in *FDPS* and confirm that the changes don’t affect any of our findings or conclusions.

The article has been corrected accordingly.

