# Supplementary material for: Genomic variations of the mevalonate pathway in porokeratosis
Source: eLife. 2015 Jul 23;4:e06322. doi: 10.7554/eLife.06322 (PMC4511816; doi:10.7554/eLife.06322)
Supplement: Figure 3—source data 1. — DOI: http://dx.doi.org/10.7554/eLife.06322.008 [file elife06322s001.pdf]

Figure 3-source data 1.

Sanger sequencing chromatograms of normal control and PK patients at 48 mutation sites in *MVK*, *PMVK*, *MVD* and *FDPS*.

No.	Genes	Mutations	Chromatograms	
			From Normal Control	From PK Patients
1	*MVK*	c.74G>T	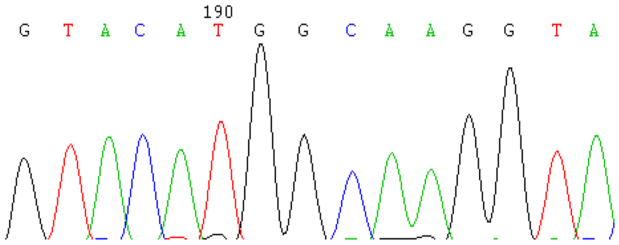	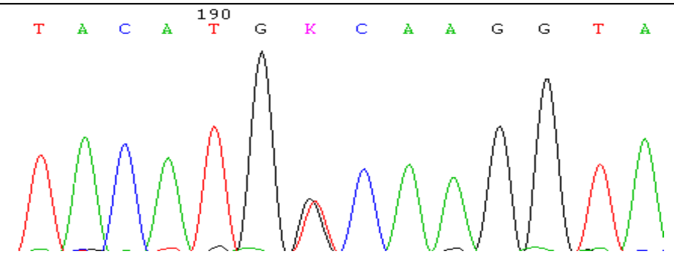
2	*MVK*	c.122T>G	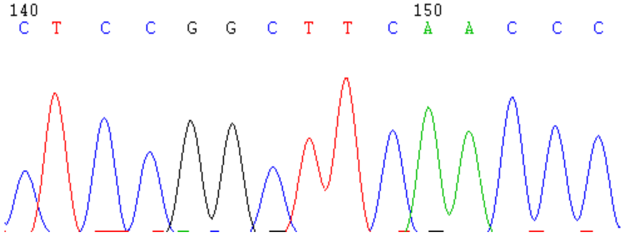	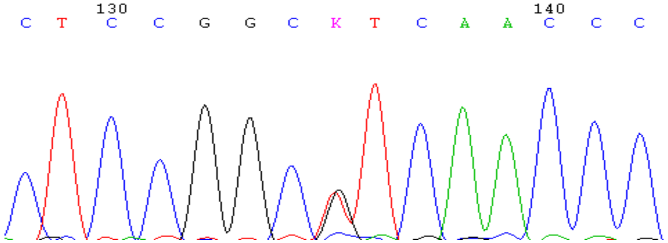
3	*MVK*	c.235G>T	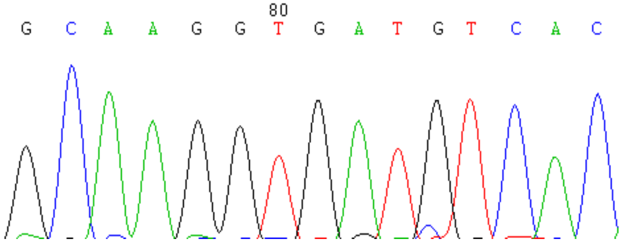	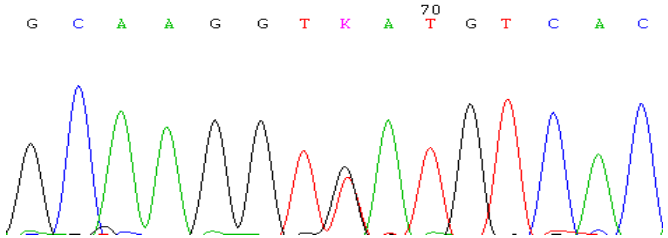
4	*MVK*	c.235G>A	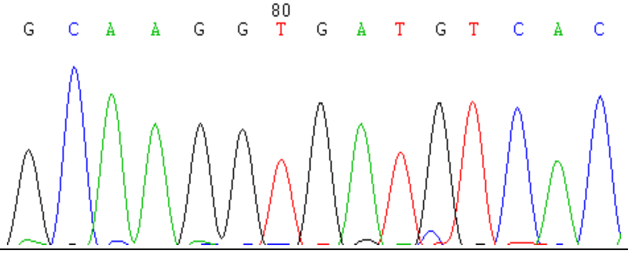	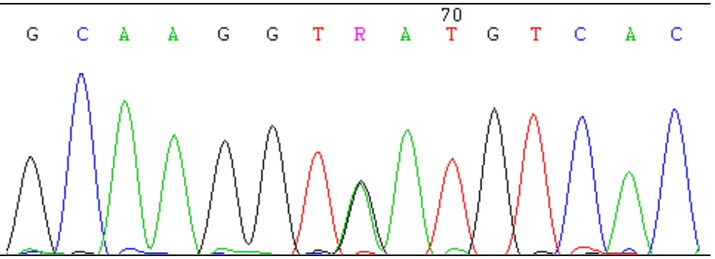

5	*MVK*	**c.254C>G**	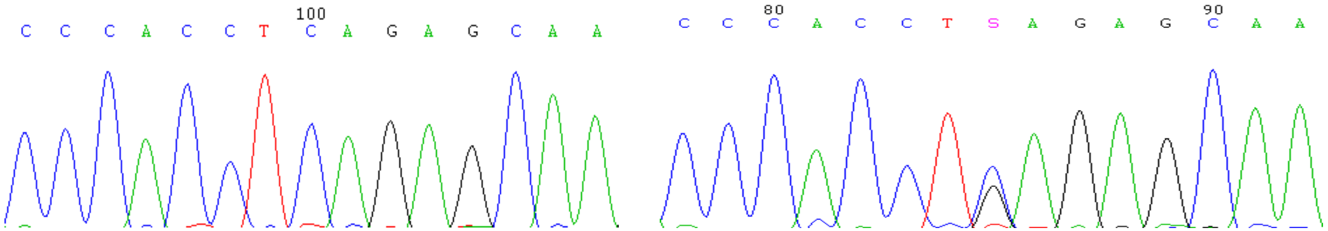
6	*MVK*	**c.371+2T>A**	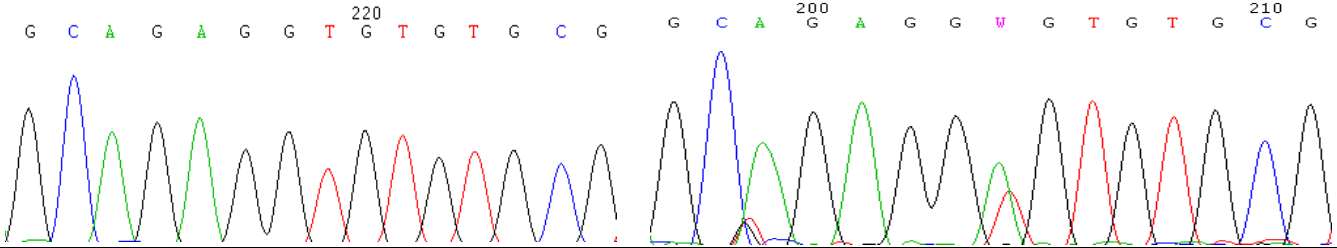
7	*MVK*	**c.395del**	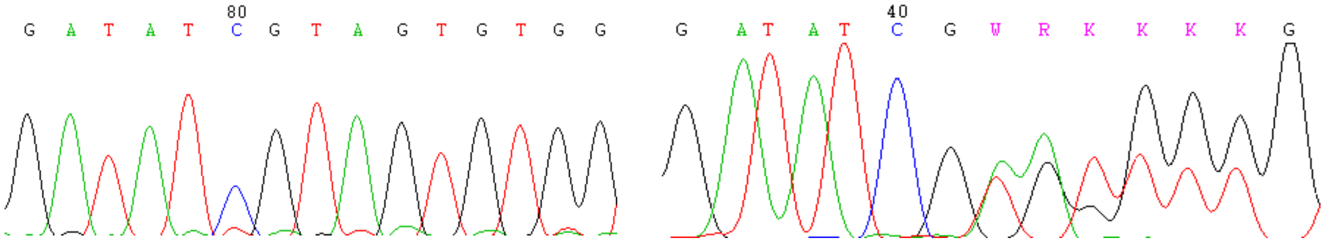
8	*MVK*	**c.417dup**	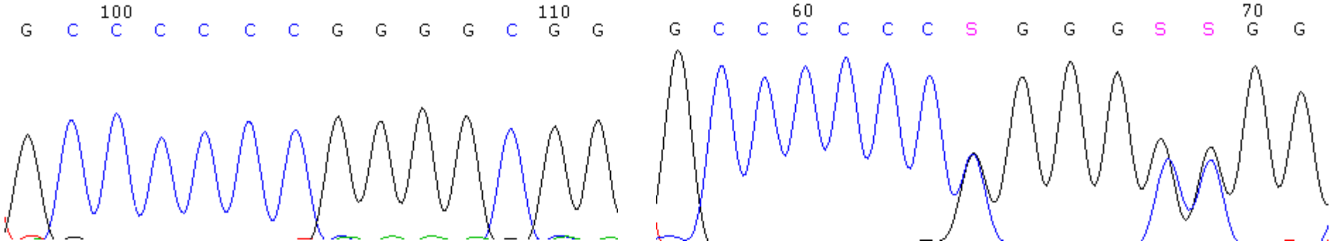

9	*MVK*	**c.437G>A**	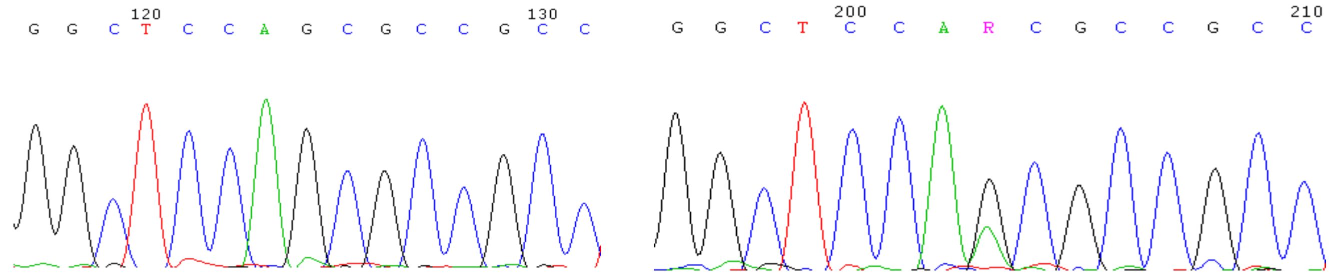
10	*MVK*	**c.451G>A**	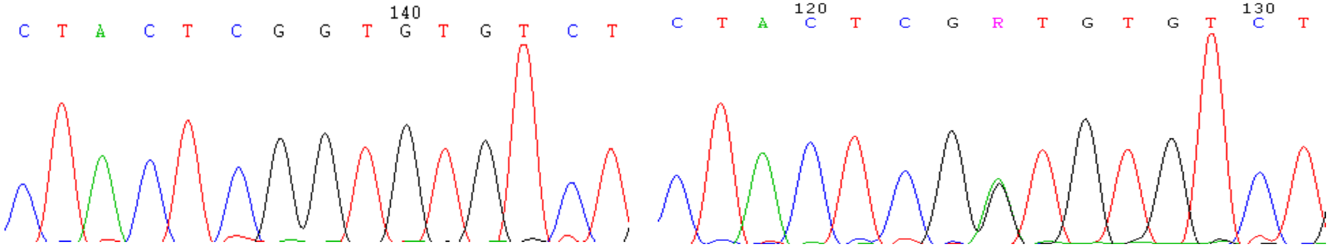
11	*MVK*	**c.481_482del**	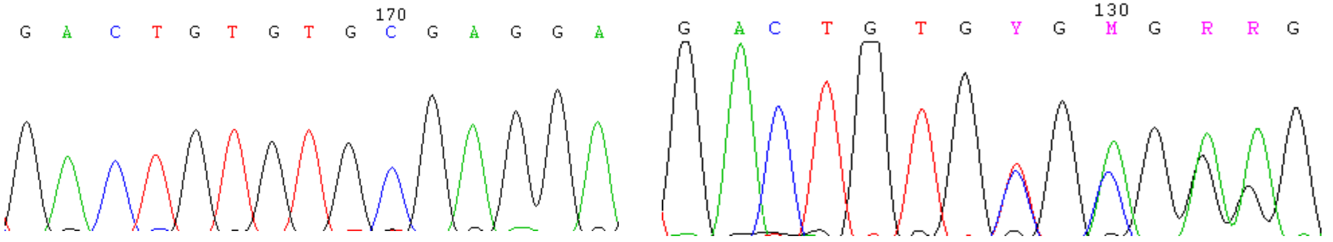
12	*MVK*	**c.604G>A**	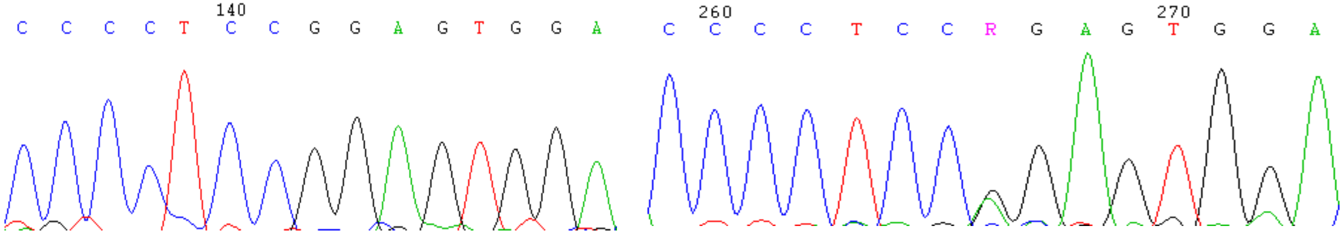
13	*MVK*	**c.605G>A**	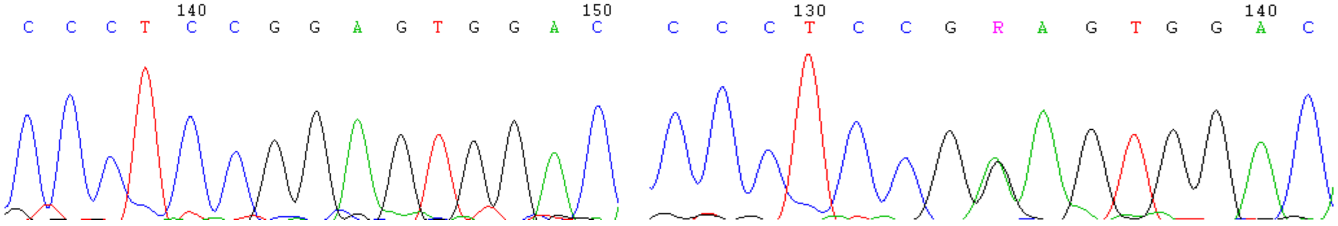

14	*MVK*	**c.650A>C**	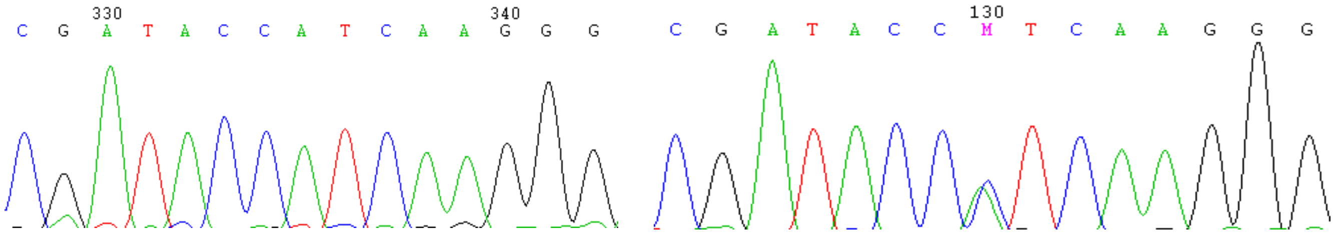
15	*MVK*	**c.671del**	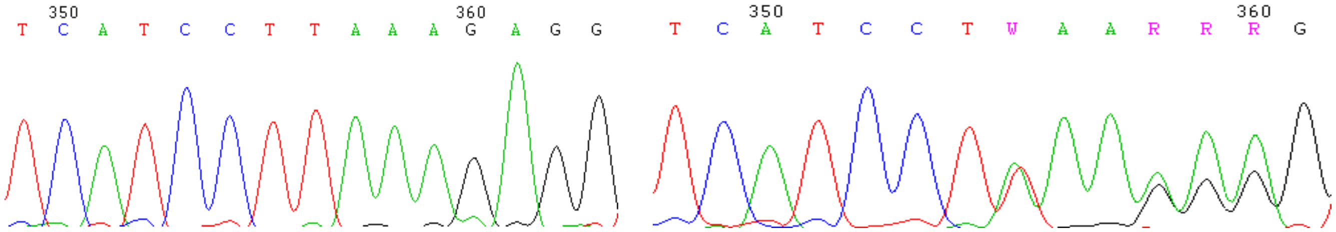
16	*MVK*	**c.710C>A**	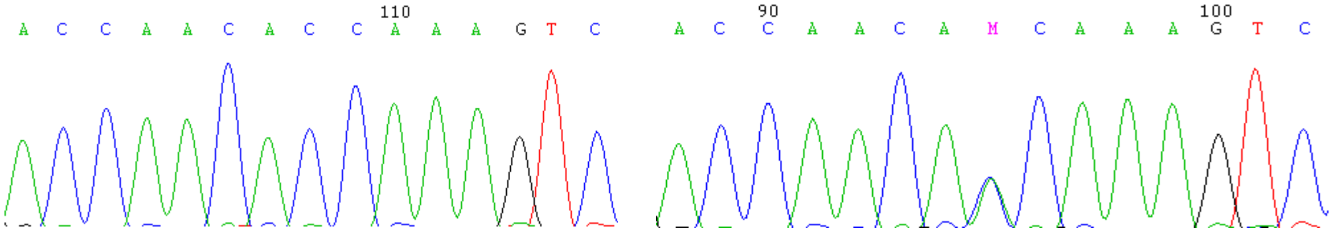
17	*MVK*	**c.902del**	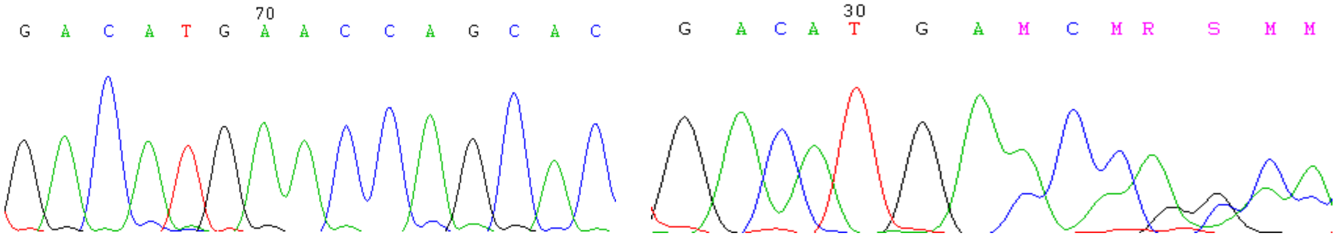
18	*MVK*	**c.904C>T**	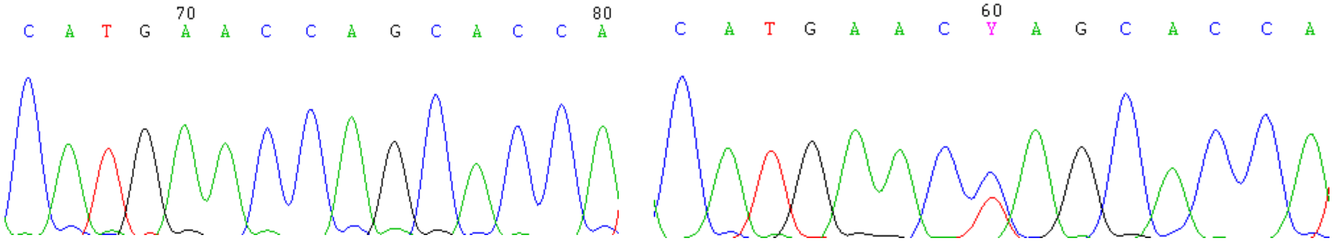

19 *MVK* **c.926G>T**

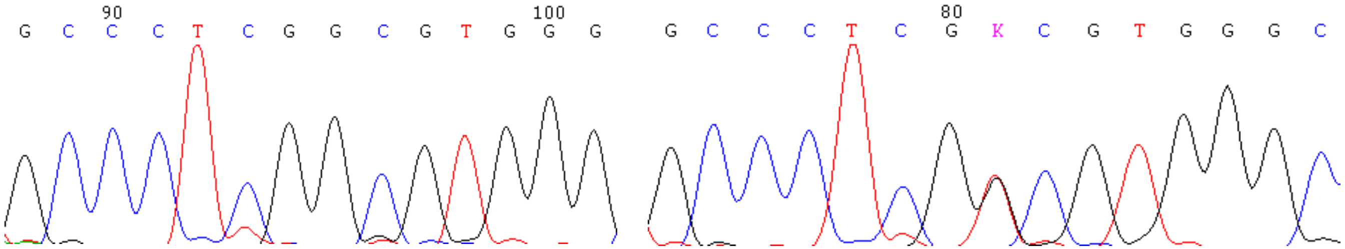

20 *MVK* **c.935A>G**

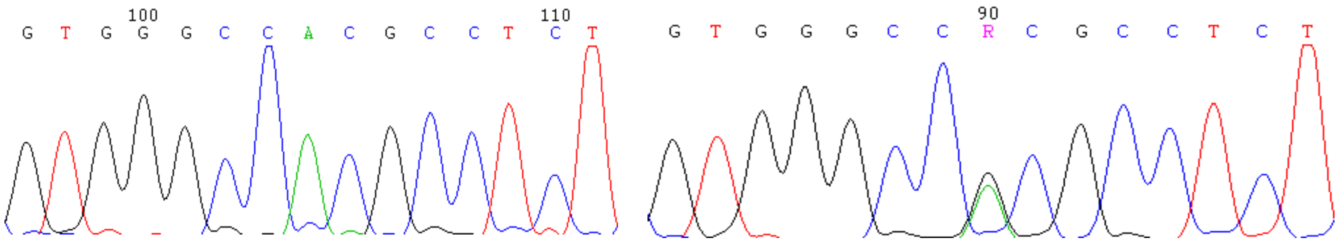

21 *MVK* **c.965C>A**

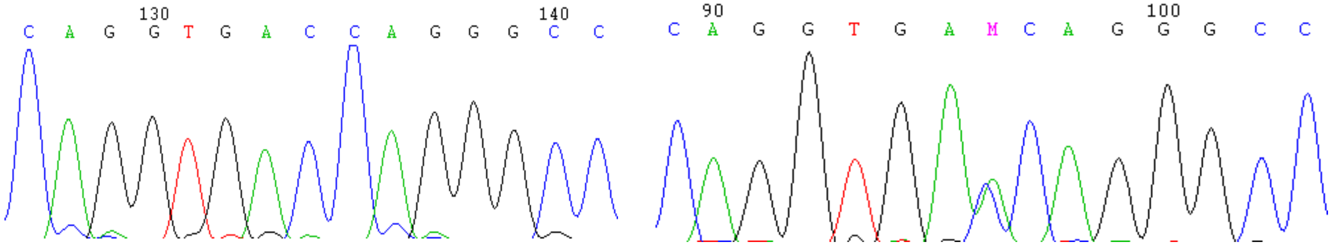

22 *MVK* **c.1012G>A**

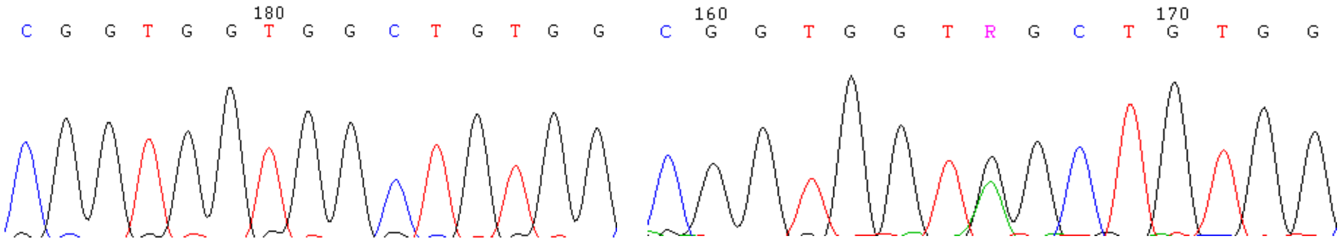

23 *MVK* **c.1024A>G**

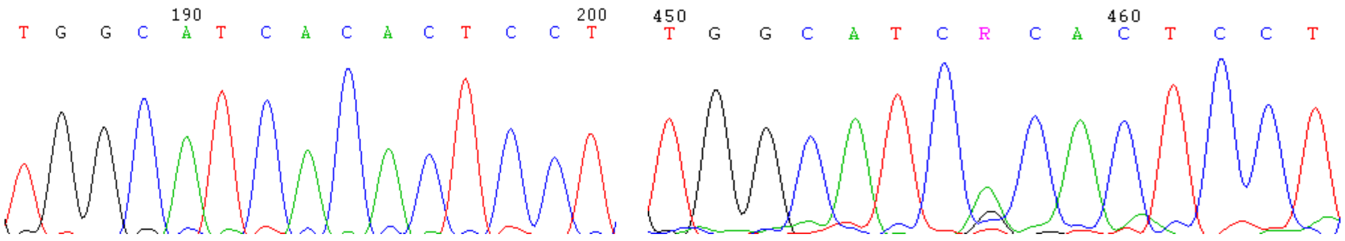

24	*MVK*	**c.1067C>G**	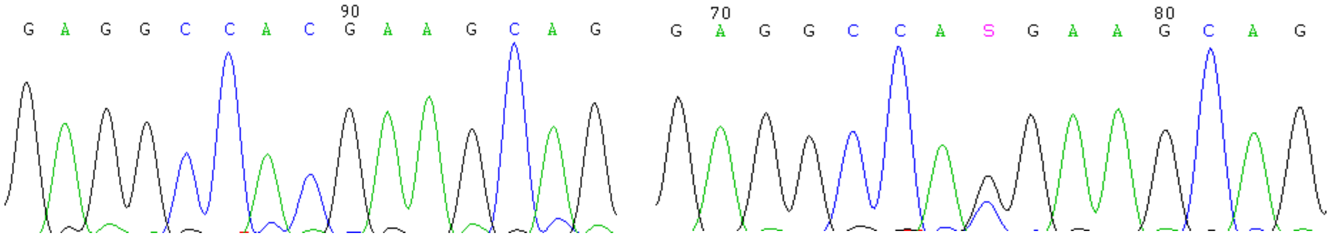
25	*MVK*	**c.1093T>A**	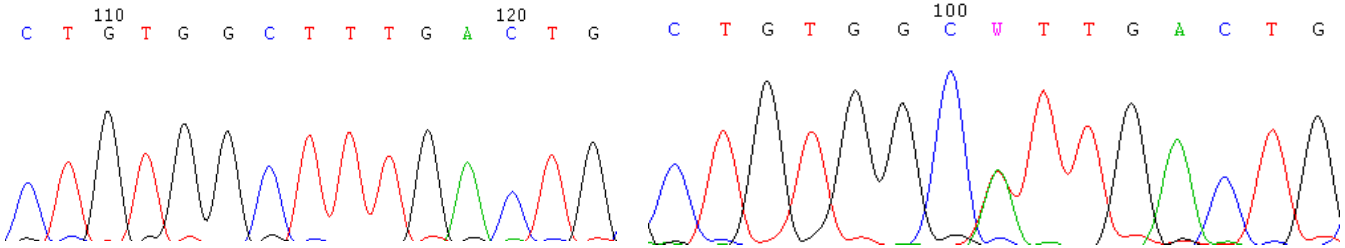
26	*MVK*	**c.1094T>C**	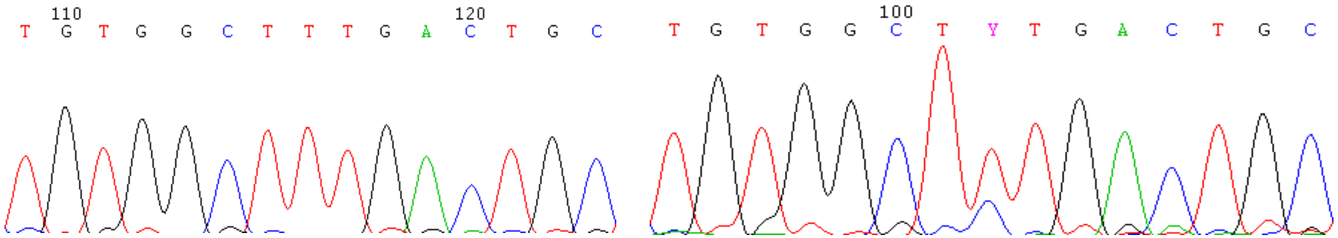
27	*MVK*	**c.1126G>A**	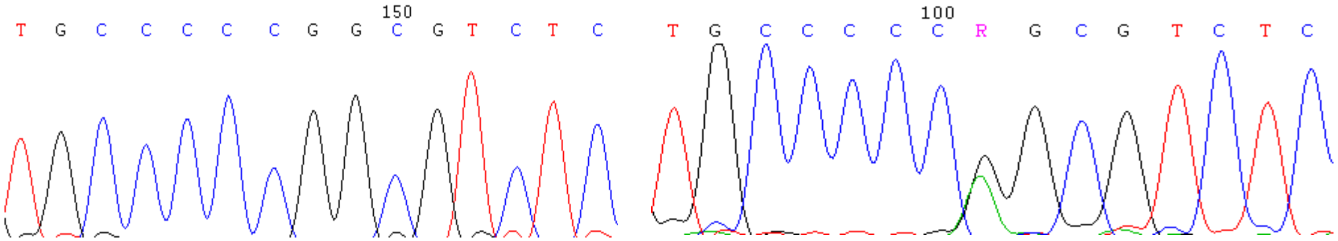

28 *PMVK* **c.1A>G**

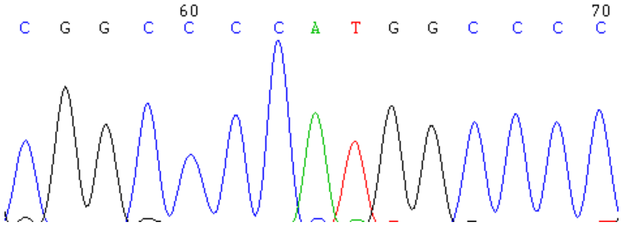

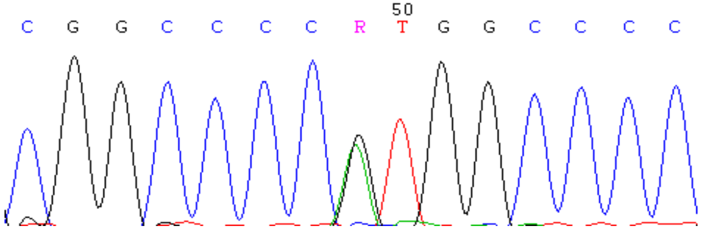

29 *PMVK* **c.94A>T**

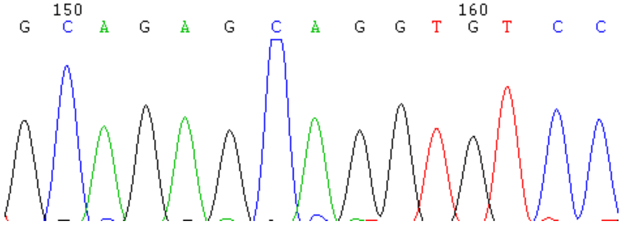

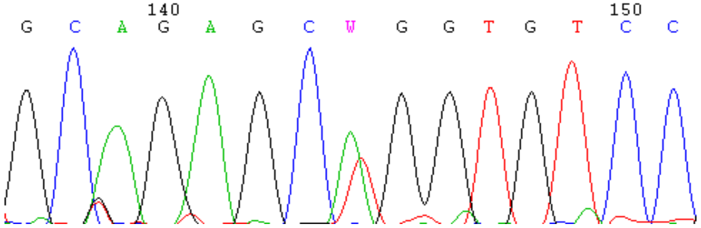

30 *PMVK* **c.205A>G**

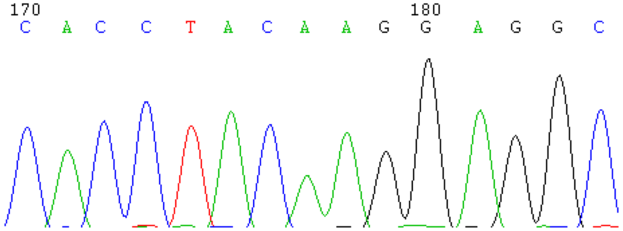

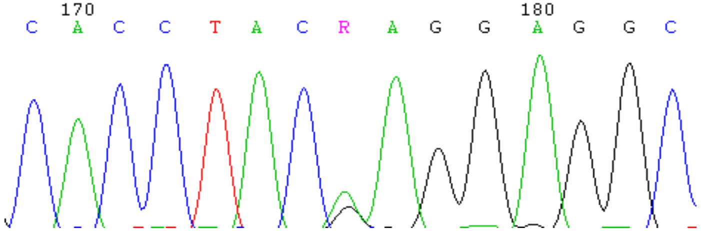

31 *PMVK* **c.312G>A**

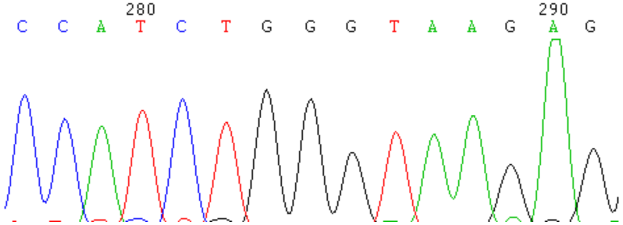

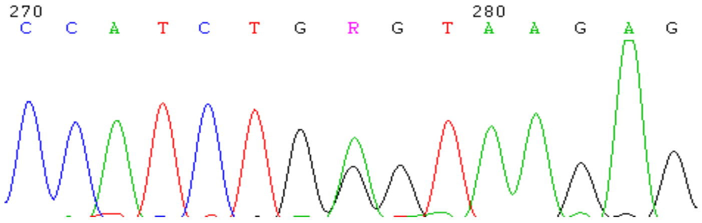

32 *PMVK* **c.412C>T**

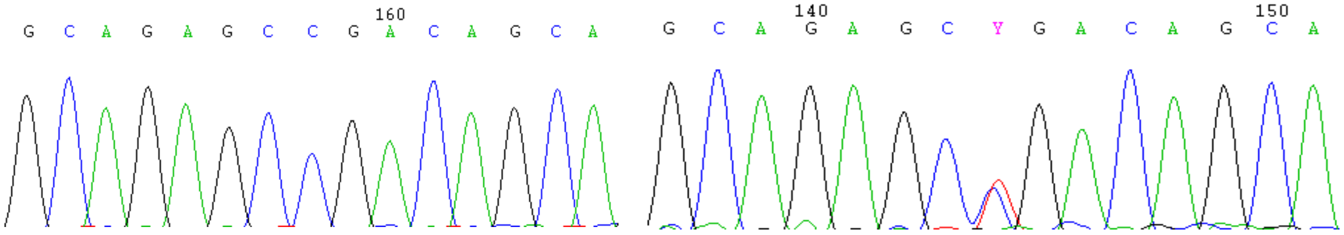

33 *PMVK* **c.550del**

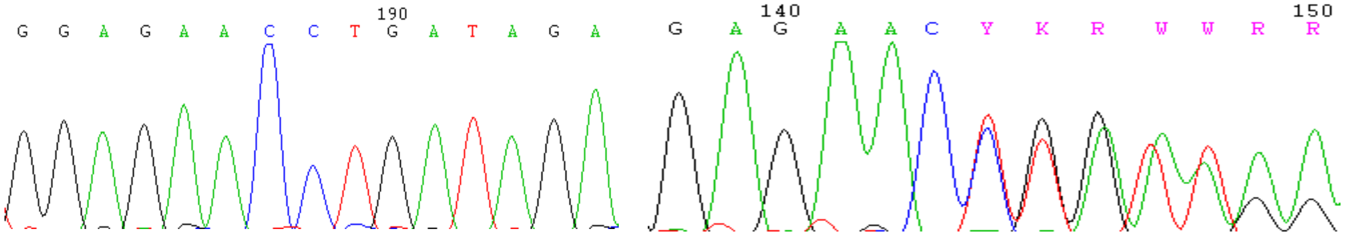

34 *MVD* **c.1A>G**

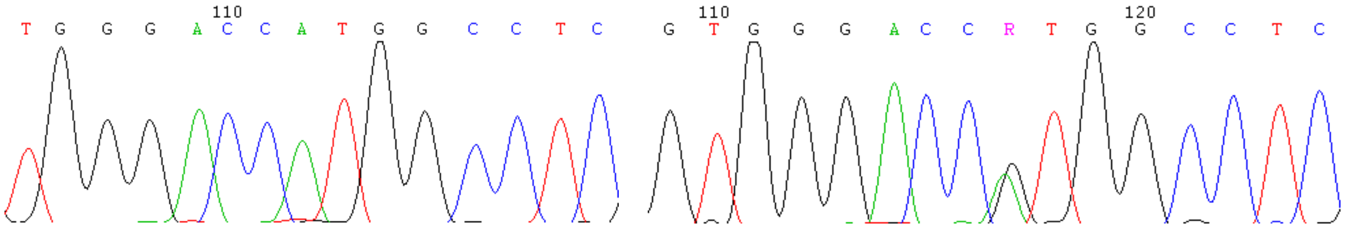

35 *MVD* **c.70+2T>G**

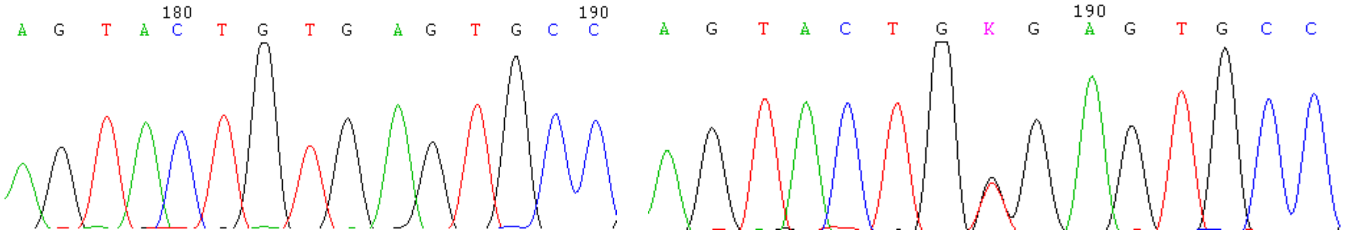

36 *MVD* **c.302C>G**

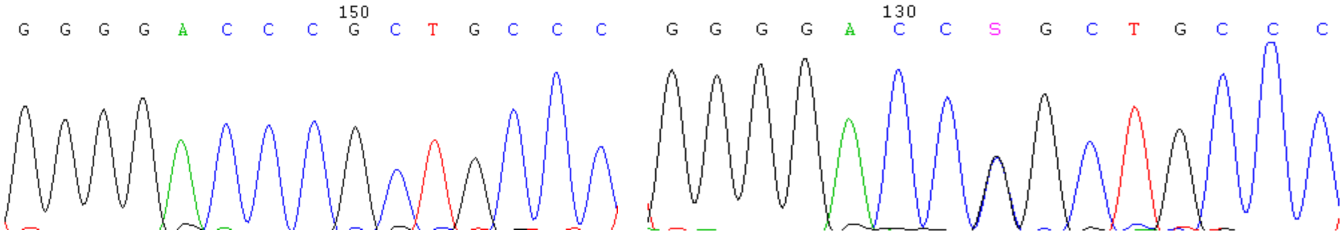

37	*MVD*	**c.383C>T**	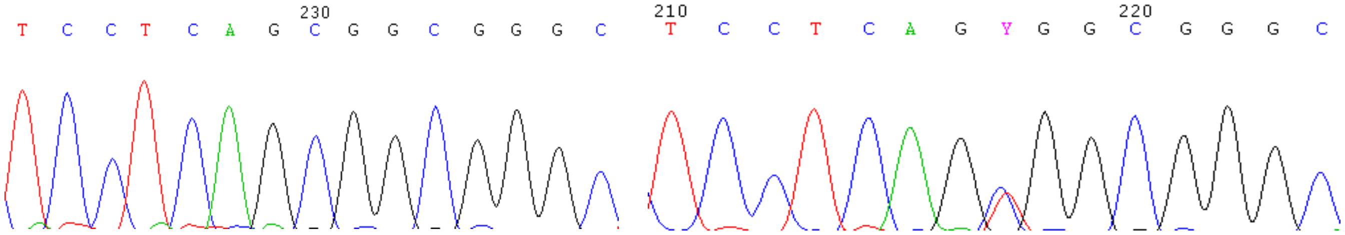
38	*MVD*	**c.482G>T**	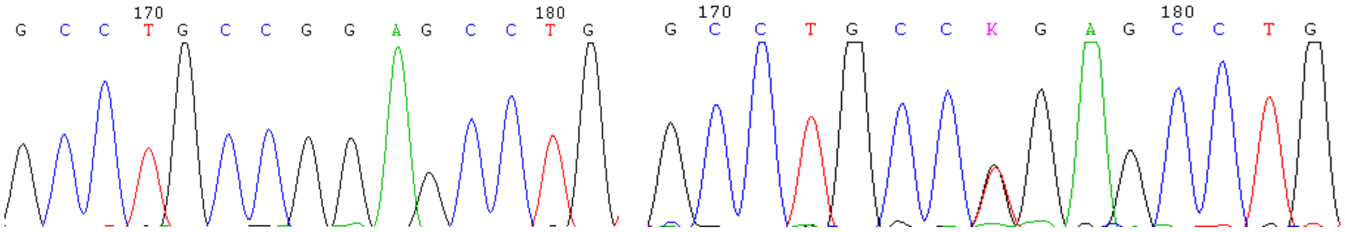
39	*MVD*	**c.482G>A**	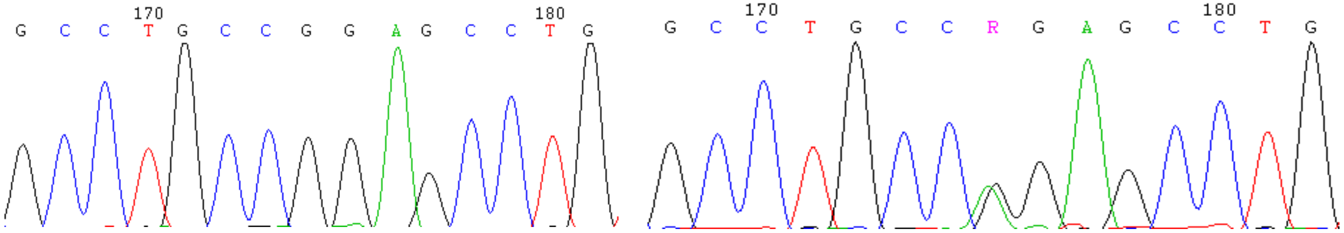
40	*MVD*	**c.678+1G>T**	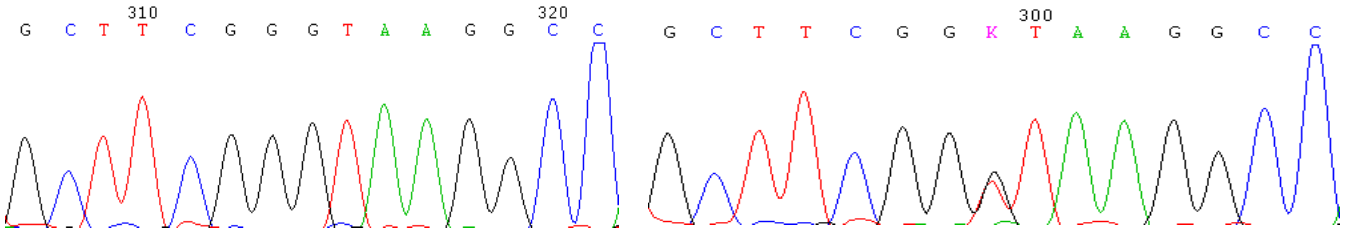
41	*MVD*	**c.682C>T**	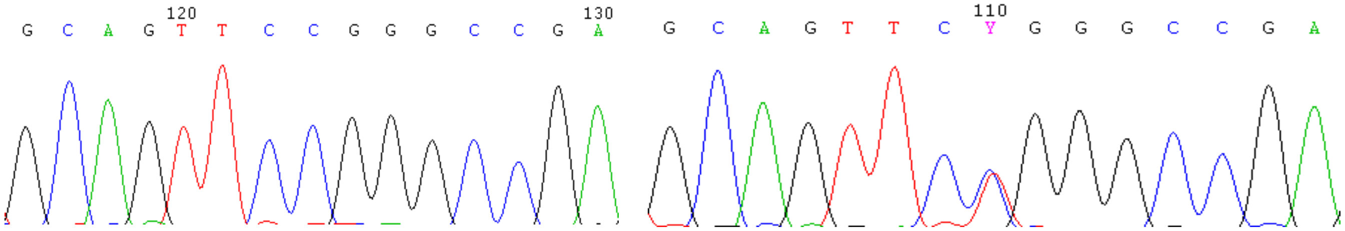

42 *MVD* **c.683G>A**

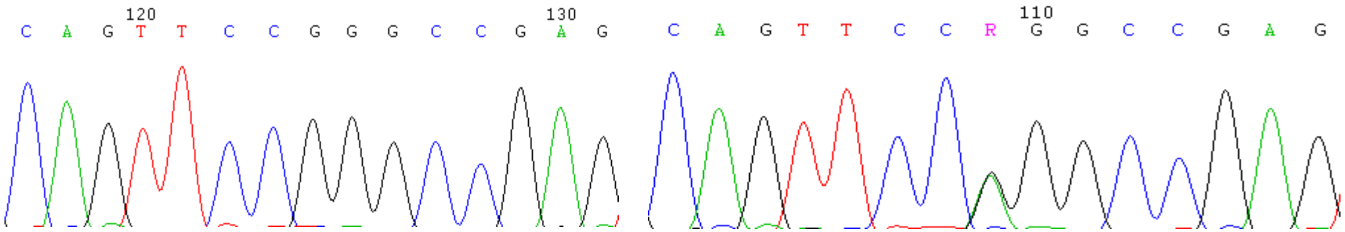

43 *MVD* **c.746T>C**

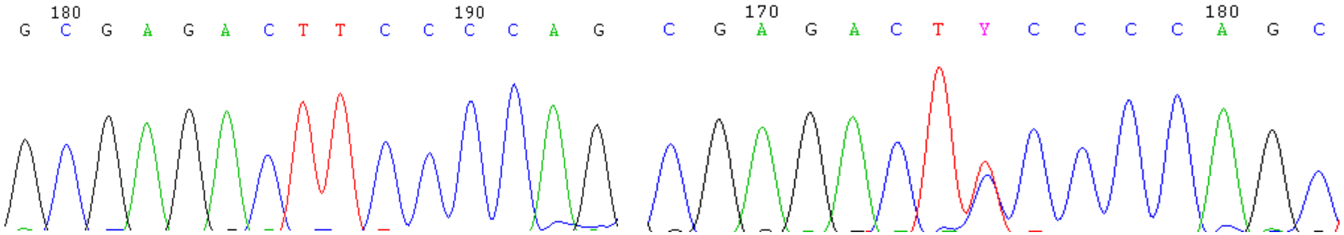

44 *MVD* **c.875A>G**

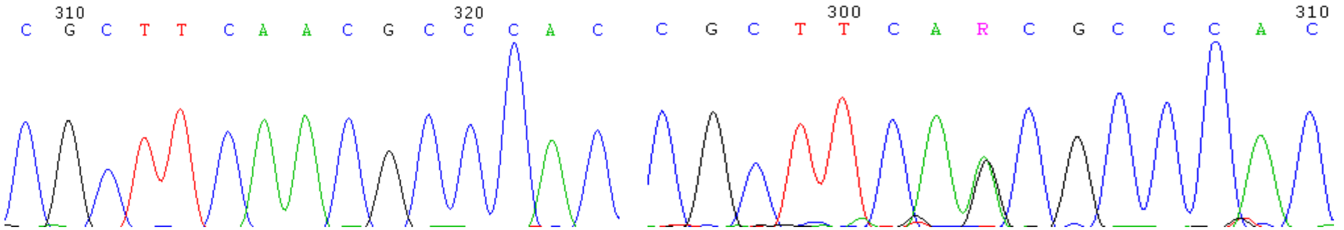

45 *MVD* **c.1111_1113 del**

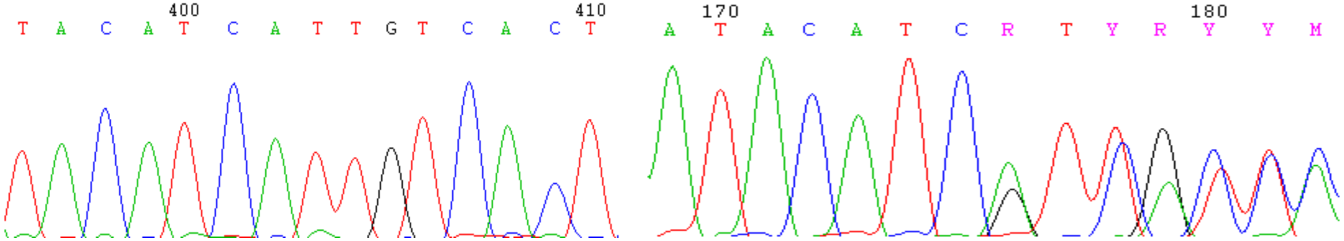

46 *MVD* **c.1126G>A**

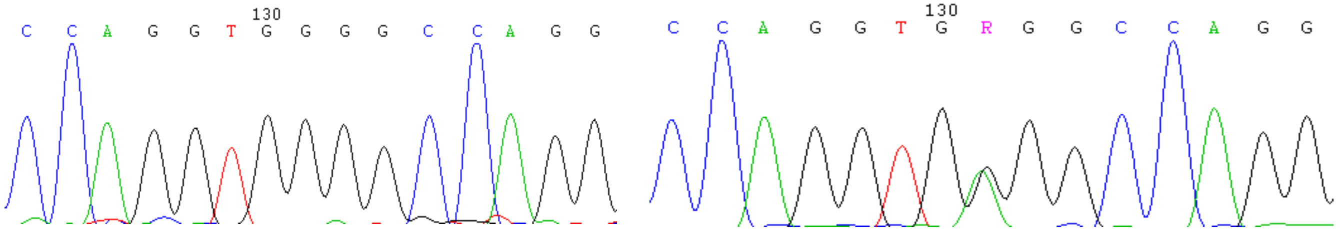

47	*FDPS*	**c.338G>A**	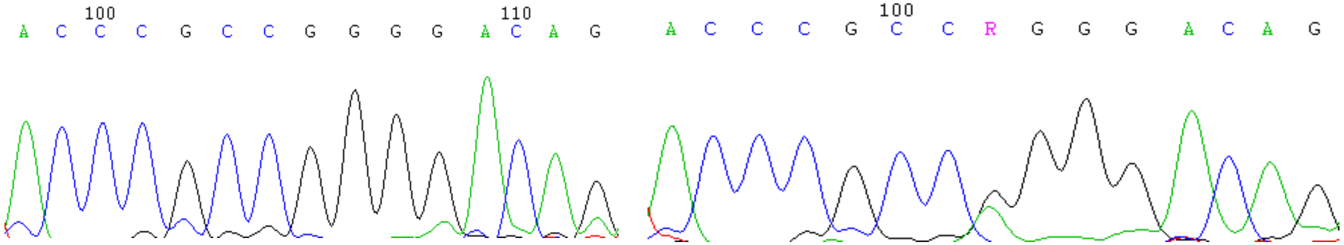
48	*FDPS*	**c.486+1G>A**	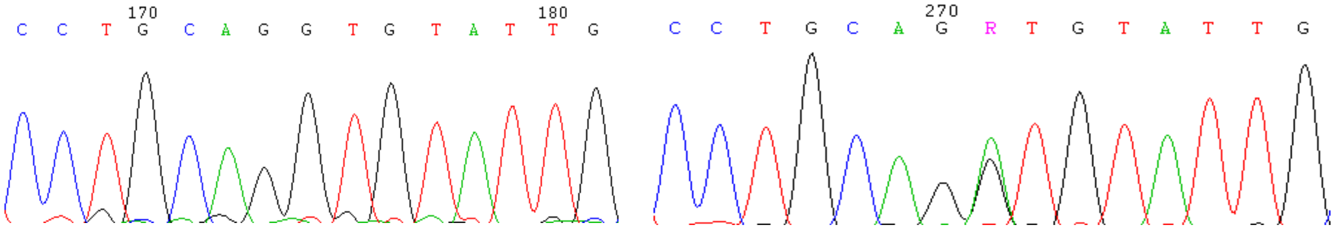
