# Supplementary material for: Genomic variations of the mevalonate pathway in porokeratosis
Source: eLife. 2015 Jul 23;4:e06322. doi: 10.7554/eLife.06322 (PMC4511816; doi:10.7554/eLife.06322)
Supplement: Supplementary file 1. — 12 non-pathogenic rare missense or nonsense single nucleotide variants (SNVs) and major reasons for exclusion. DOI: http://dx.doi.org/10.7554/eLife.06322.017 [file elife06322s002.docx]

**Supplementary file 1.**

**12 nonpathogenic rare missense or nonsense SNVs and major reasons for exclusion.**

|  | **Gene** | **Mutation** | **Predicted protein alteration** | **Mutation type** | **Familial case** | **Sporadic case** | **dbSNP**  **(MAF)** | **SIFT**  **Score** | **Poly**  **-Phen**  **Score** | **Mutation**  **Taster**  **Score** | **In 270 controls (p-value)** | **Major reason for exclusion** |
| --- | --- | --- | --- | --- | --- | --- | --- | --- | --- | --- | --- | --- |
| 1 | ACAT1 | c.163T>A | p.Phe55Ile | Missense |  | S-31 | 0.02 | 0.999 | 0.999616 | 0 | 0.02 | S-31 also carrying a common MVD mutation (c.746T>C) much more likely to be pathogenic |
| 2 | ACAT1 | c.622C>T | p.Arg208* | Nonsense |  | S-42 | 1 | 0.733221 | 1 | 0 | 1 | No solid evidence to support mutations in ACAT1 to be pathogenic for PK |
| 3 | ACAT2 | c.851C>G | p.Ser284Cys | Missense | F-24; F-61 |  | 0 | 0.189 | 0.914044 | 7 (p=0.48) | 0 | Identified in controls with no significant difference |
| 4 | HMGCS2 | c.175C>A | p.Leu59Met | Missense | F-26 | S-57 | 0.55 | 0 | 0.705514 | 3 (p=0.74) | 0.55 | Identified in controls with no significant difference |
| 5 | HMGCS2 | c.791G>A | p.Arg246Gln | Missense |  | S-33 | 0.54 | 0.01 | 0.712758 | 0 | 0.54 | S-33 also carrying a MVD mutation(c.1111_1113del) more likely to be pathogenic |
| 6 | HMGCS2 | c.1312T>A | p.Leu438Met | Missense |  | S-40 | 0.07 | 0 | 0.921431 | 0 | 0.07 | S-40 also carrying a common MVD mutation (c.746T>C) much more likely to be pathogenic |
| 7 | HMGCR | c.932A>C | p.Tyr311Ser | Missense | F-26 | S-5,S-69 | 0.03 | 0.285 | 0.999912 | 12 (p=0.10) | 0.03 | Identified in controls with no significant frequency difference |
| 8 | MVD | c.182C>A | p.Thr61Asn | Missense |  | S-47 |  | 0.001 | 0.816354 | 2 (p=0.99) |  | Identified in controls with no significant frequency difference |
| 9 | MVD | c.629G>A | p.Gly210Asp | Missense |  | S-24 | 0 | 0.077 | 0.959602 | 2 (p=0.99) | 0 | Identified in controls with no significant frequency difference |
| 10 | MVD | c.985G>A | p.Gly329Ser | Missense |  | S-12 |  | 0 | 0.000093 | 2 (p=0.99) |  | Identified in controls with no significant frequency difference |
| 11 | IDI2 | c.419C>G | p.Ser140* | Nonsense |  | S-35 | 0.07 | 0.729644 | 1 | 0 | 0.07 | S-35 also carrying a PMVK mutation (c.550del) more likely to be pathogenic |
| 12 | IDI2 | c.631C>T | p.His211Tyr | Missense |  | S-15 | 0.21 | 0.001 | 0.023284 | 0 | 0.21 | No solid evidence to support mutations in *IDI2* to be pathogenic for PK |
