# Supplementary material for: Genomic variations of the mevalonate pathway in porokeratosis
Source: eLife. 2015 Jul 23;4:e06322. doi: 10.7554/eLife.06322 (PMC4511816; doi:10.7554/eLife.06322)
Supplement: Supplementary file 2. — Characterization of 51 mutations identified in 113 of the 134 porokeratosis (PK) patients. DOI: http://dx.doi.org/10.7554/eLife.06322.018 [file elife06322s003.docx]

**Supplementary file 2.**

**Characterization of 51 mutations identified in 113 of the 134 PK patients.**

| No. | Gene | Mutation | Exon | Predicted protein alternation | Mutation  type | Cases | | dbSNP  (MAF) | SIFT  Score | Poly  -Phen  Score | Mutation  Taster  Score | In 270 controls (p-value) | Reported in PK |
| --- | --- | --- | --- | --- | --- | --- | --- | --- | --- | --- | --- | --- | --- |
|  |  |  |  |  |  | Familial (affected / unaffected ) | Sporadic |  |  |  |  |  |  |
| 1 | *MVK* | c.74G>T | 2 | p.Gly25Val | Missense | F-24(16/8) |  |  | 0 | 1 | 0.999999 | 0 |  |
| 2 | *MVK* | c.122T>G | 3 | p.Leu41Arg | Missense | F-44(1/0) |  |  | 0 | 0.984 | 0.999998 | 0 |  |
| 3 | *MVK* | c.235G>A | 4 | p.Asp79Asn | Missense | F-28(1/0); |  |  | 0.25 | 0.021 | 0.958465 | 0 |  |
| 4 | *MVK* | c.235G>T | 4 | p. Asp79Tyr | Missense | F-50(1/0) |  |  | 0.01 | 0.847 | 0.995562 | 0 |  |
| 5 | *MVK* | c.254C>G | 4 | p.Ser85* | Nonsense | F-30(1/0) |  |  | 0.42 | 0.505671 | 1 | 0 |  |
| 6 | *MVK* | c.371+2T>A | 4 | p.Glu76Glyfs*9 | Splicing defect | F-42(1/0) ^#^ |  |  |  |  |  | 0 |  |
| 7 | *MVK* | c.395del | 5 | p.Val132Glufs*27 | Indel | F-29(1/0); F-31(1/0)^#^ | S-30^#^ |  |  |  |  | 0 | *Li et al., 2014* |
| 8 | *MVK* | c.417dup | 5 | p.Gly140Argfs*47 | Indel |  | S-50 |  |  |  |  | 0 | *Li et al., 2014* |
| 9 | *MVK* | c.437G>A | 5 | p.Ser146Asn | Missense | F-46(1/0) |  |  | 0 | 0.999 | 1 | 0 |  |
| 10 | *MVK* | c.451G>A | 5 | p.Val151Met | Missense | F-55(1/0) |  |  | 0.01 | 0.938 | 0.999995 | 0 |  |
| 11 | *MVK* | c.481_482  del | 5 | p.Cys161Argfs*25 | Indel |  | S-38 |  |  |  |  | 0 | *Li et al., 2014* |
| 12 | *MVK* | c.604G>A | 6 | p.Gly202Arg | Missense | F-19(8/9) | S-67 | rs104895301(NA) | 0 | 1 | 1 | 0 | *Li et al., 2014* |
| 13 | *MVK* | c.605G>A | 6 | p.Gly202Glu | Missense |  | S-57 |  | 0 | 1 | 1 | 0 |  |
| 14 | *MVK* | c.650A>C | 7 | p.His217Pro | Missense | F-15(4/2) |  |  | 0 | 0.002 | 0.000739 | 0 |  |
| 15 | *MVK* | c.671del | 7 | p.Leu224* | Indel |  | S-26^#^ |  |  |  |  | 0 |  |
| 16 | *MVK* | c.710C>A | 8 | p.Thr237Asn | Missense | F-13(7/1) |  |  | 0 | 0.992 | 0.999254 | 0 |  |
| 17 | *MVK* | c.902del | 10 | p.Asn301Thrfs*5 | Indel |  | S-16 |  |  |  |  | 0 |  |
| 18 | *MVK* | c.904C>T | 10 | p.Gln302* | Nonsense | F-32(1/0) ^#^ |  |  | 0 | 0.73522 | 1 | 0 |  |
| 19 | *MVK* | c.926G>T | 10 | p.Gly309Val | Missense |  | S-39 |  | 0 | 1 | 1 | 0 |  |
| 20 | *MVK* | c.935A>G | 10 | p.His312Arg | Missense | F-1(17/14); F-43(1/0)^#^; F-59(4/1) |  |  | 0 | 1 | 0.999998 | 0 | *Li et al., 2014* |
| 21 | *MVK* | c.965C>A | 10 | p.Thr322Asn | Missense | F-54(2/0) |  |  | 0.01 | 0.979 | 0.99999 | 0 |  |
| 22 | *MVK* | c.1012G>A | 10 | p.Gly338Ser | Missense |  | S-11 |  | 0 | 1 | 0.999999 | 0 |  |
| 23 | *MVK* | c.1024A>G | 10 | p.Thr342Ala | Missense | F-2(17/20) |  |  | 0.14 | 0.591 | 0.999641 | 0 |  |
| 24 | *MVK* | c.1067C>G | 11 | p.Thr356Arg | Missense |  | S-32 |  | 0.03 | 0.661 | 0.701278 | 0 |  |
| 25 | *MVK* | c.1093T>A | 11 | p.Phe365Ile | Missense | F-38(1/0) ^#^ |  |  | 0.01 | 1 | 0.999999 | 0 |  |
| 26 | *MVK* | c.1094T>C | 11 | p.Phe365Ser | Missense | F-16(1/0); F-58(1/0) |  |  | 0 | 1 | 0.999999 | 0 | *Li et al., 2014* |
| 27 | *MVK* | c.1126G>A | 11 | p.Gly376Ser | Missense | F-22(4/0); | S-22; S47 |  | 0 | 0.996 | 0.999999 | 0 | *Li et al., 2014* |
| 28 | *MVK* | c.-1880_527+533del | 1-5 | p.? | Large deletion | F-26(1/0); F-40(1/0) | S-21;S-69 |  |  |  |  | 0 |  |
| 29 | *PMVK* | c.1A>G | 1 | p.Met1? | Start codon |  | S-17 |  | 0 | 0.969 | 1 | 0 |  |
| 30 | *PMVK* | c.94A>T | 1 | p.Arg32* | Nonsense |  | S-55 |  | 0.16 | 0.735024 | 1 | 0 |  |
| 31 | *PMVK* | c.205A>G | 3 | p.Lys69Glu | Missense | F-52(1/0) |  |  | 0 | 0.937 | 0.999577 | 0 |  |
| 32 | *PMVK* | c.312G>A | 3 | p.Trp104* | Nonsense |  | S-58 |  | 0.11 | 0.735424 | 1 | 0 |  |
| 33 | *PMVK* | c.412C>T | 4 | p.Arg138* | Nonsense | F-56(1/0); F-60(1/0) | S-19; S-71 |  | 1 | 0.735406 | 1 | 0 |  |
| 34 | *PMVK* | c.550del | 5 | p.Leu184* | Indel |  | S-35 |  |  |  |  | 0 |  |
| 35 | *MVD* | c.1A>G | 1 | p.Met1? | Start codon | F-11(4/3); F-23(6/3) |  |  |  | 0.992 | 1 | 0 |  |
| 36 | *MVD* | c.70+2T>G | 2 | p.? | Splicing defect | F-39(1/0) |  |  |  |  |  | 0 |  |
| 37 | *MVD* | c.302C>G | 4 | p.Pro101Arg | Missense |  | S-62 | rs200033380  (0.0005) |  | 0 | 0.749732 | 0 |  |
| 38 | *MVD* | c.383C>T | 4 | p.Ala128Val | Missense |  | S-61 |  |  | 0.998 | 0.999994 | 0 |  |
| 39 | *MVD* | c.482G>A | 5 | p.Arg161Gln | Missense |  | S-72 | rs144010349(NA) |  | 1 | 0.999981 | 0 |  |
| 40 | *MVD* | c.482G>T | 5 | p.Arg161Leu | Missense |  | S-13 |  |  | 1 | 0.999851 |  |  |
| 41 | *MVD* | c.678+1G>T | 7 | p.? | Splicing defect | F-27(1/0) |  |  |  |  |  | 0 |  |
| 42 | *MVD* | c.682C>T | 7 | p.Arg228Trp | Missense |  | S-1 |  | 0 | 1 | 0.999978 | 0 |  |
| 43 | *MVD* | c.683G>A | 7 | p.Arg228Gln | Missense |  | S-62 |  | 0.01 | 0.999 | 0.999851 | 0 |  |
| 44 | *MVD* | c.746T>C | 7 | p.Phe249Ser | Missense | F-3(12/12); F-5(4/2); F-8(1/0); F-9(1/0); F-10(1/1); F-17(1/0); F-21(1/1); F-25(2/2); F-28(1/0); F-34(1/0); F-35(1/0) ^#^; F-45(1/0); F-48(2/0); F-51(1/0); F-53(1/0); F-57(1/0); F20(1/0) | S-3; S-5;  S-9; S-10; S-28^#^; S-29; S-31; S-37; S-40; S-41; S-43; S-52; S-53; S-60; S-63; S-65 |  |  | 1 | 0.999989 | 1 (p=0.00)* |  |
| 45 | *MVD* | c.875A>G | 7 | p.Asn292Ser | Missense | F-7(3/0); F-12(1/0); F-14(1/0); F-18(1/0); F-33(1/0)^#^; F-36(1/0); F-37(1/0)^#^; F-41(1/0); F-49(1/0); F-61(1/0) | S-6; S-7; S-18; S-25; S-51; S-64; S-73   \|  \| \| --- \| |  | 0 | 0.999 | 0.999896 | 0 |  |
| 46 | *MVD* | c.1111_1113del | 9 | p.Ile371del | Deletion |  | S-33; S-66 |  |  |  |  | 0 |  |
| 47 | *MVD* | c.1126G>A | 10 | p.Gly376Arg | Missense |  | S-68 |  | 0 | 0.996 | 0.999999 | 0 |  |
| 48 | *FDPS* | c.-1129_141+994del | 1-2 | p.? | Large deletion |  | S-56 |  |  |  |  | 0 |  |
| 49 | *FDPS* | c.283-1776_649-143del | 4-7 | p.? | Large deletion | F-6(2/1) |  |  |  |  |  | 0 |  |
| 50 | *FDPS* | c.338G>A | 4 | p.Arg113Gln | Missense | F-47(1/1) |  |  | 0.05 | 0.872 | 0.999167 | 0 |  |
| 51 | *FDPS* | c.486+1G>A | 5 | p.Ser163_Lys353delins13 | Splicing defect |  | S-36^#^ |  |  |  |  | 0 |  |

Notes:

1. There were 28 mutations in *MVK*, 6 mutations in *PMVK*, 13 mutations in *MVD* and 4 mutations in *FDPS*, which were identified in 113 of the 134 index PK patients. As for the familial cases, the number of affected and unaffected members were listed in parentheses. SIFT, PolyPhen-2, and MutationTaster were used to assess the conservation of the the missense mutations.
2. ^#^ Pairwise LT and NNS were dissected.
3. *c.746T>C (p.Phe249Ser) in *MVD* was identified in one 22 year-old male, who took routine medical examination in the hospital. This male had no PK lesions till now and no familial history of skin diseases. Because this mutation showed the highest frequency of occurence in PK, the p-value of this mutation showed significant difference.
