# Supplementary material for: Genomic variations of the mevalonate pathway in porokeratosis
Source: eLife. 2015 Jul 23;4:e06322. doi: 10.7554/eLife.06322 (PMC4511816; doi:10.7554/eLife.06322)
Supplement: Supplementary file 3. — Mutant to wild allelic ratio measurements in both genomic DNA and complementary DNA of 13 pairwise tissue sets from 12 patients. DOI: http://dx.doi.org/10.7554/eLife.06322.019 [file elife06322s004.docx]

**Supplementary file 3.**

**Mutant to wild allelic ratio measurements in both genomic DNA and complementary DNA of 13 pairwise tissue sets from 12 patients.**

| **No.** | **Patient** | **Sex** | **Age** | **Tissue Location** | | **Mutation** | **Sample** | **AEI Measurements (M/W)** | | | | **Mean** | **STD** | ***p* value1** | ***p* value2** |
| --- | --- | --- | --- | --- | --- | --- | --- | --- | --- | --- | --- | --- | --- | --- | --- |
|  |  |  |  |  |  |  |  | **A1** | **A2** | **B1** | **B2** |  |  |  |  |
| 1 | F-42 | M | 60 | | Buttock | MVK:c.371+2T>A | NNS-gDNA | 0.91 | 1.06 |  |  | 0.98 | 0.11 |  |  |
|  |  |  |  | |  |  | LT-gDNA | 1.34 | 1.24 |  |  | 1.29 | 0.07 | 0.08320 |  |
|  |  |  |  | |  |  | NNS-cDNA | 0.89 | 0.81 | 0.52 | 0.62 | 0.71 | 0.17 |  |  |
|  |  |  |  | |  |  | LT-cDNA | 0.65 | 0.81 | 1.36 | 0.81 | 0.91 | 0.31 |  | 0.80449 |
| 2 | F-31 | M | 39 | | Buttock | MVK:395delT | NNS-gDNA | 1.00 | 1.06 |  |  | 1.03 | 0.05 |  |  |
|  |  |  |  | |  |  | LT-gDNA | 3.27 | 3.41 |  |  | 3.34 | 0.10 | 0.00117 |  |
|  |  |  |  | |  |  | NNS-cDNA | 0.13 | 0.23 | 0.14 | 0.56 | 0.27 | 0.20 |  |  |
|  |  |  |  | |  |  | LT-cDNA | 3.05 | 2.72 | 3.19 | 3.90 | 3.21 | 0.50 |  | 0.00126 |
| 3 | S-30 | M | 35 | | Buttock | MVK:c.395delT | NNS-gDNA | 1.06 | 0.86 |  |  | 0.96 | 0.14 |  |  |
|  |  |  |  | |  |  | LT-gDNA | 1.24 | 1.13 |  |  | 1.19 | 0.08 | 0.17987 |  |
|  |  |  |  | |  |  | NNS-cDNA | 0.18 | 0.16 | 0.29 | 0.28 | 0.23 | 0.01 |  |  |
|  |  |  |  | |  |  | LT-cDNA | 2.85 | 1.97 |  |  | 2.41 | 0.63 |  | 0.00137 |
| 4 | S-26 | M | 29 | | Buttock | MVK:c.671delT | NNS-gDNA | 1.02 | 1.05 |  |  | 1.04 | 0.02 |  |  |
|  |  |  |  | |  |  | LT-gDNA | 1.23 | 1.22 |  |  | 1.23 | 0.01 | 0.00959 |  |
|  |  |  |  | |  |  | NNS-cDNA | 0.16 | 0.17 | 0.27 | 0.17 | 0.20 | 0.05 |  |  |
|  |  |  |  | |  |  | LT-cDNA | 2.47 | 2.44 | 2.40 | 2.56 | 2.47 | 0.07 |  | 1.43E-06 |
| 5 | F-32 | F | 50 | | Buttock | MVK:c.904C>T | NNS-gDNA | 1.33 | 1.20 |  |  | 1.27 | 0.09 |  |  |
|  |  |  |  | |  |  | LT-gDNA | 1.14 | 1.06 |  |  | 1.10 | 0.05 | 0.15263 |  |
|  |  |  |  | |  |  | NNS-cDNA | 0.33 | 0.32 | 0.31 | 0.34 | 0.33 | 0.01 |  |  |
|  |  |  |  | |  |  | LT-cDNA | 2.76 | 2.52 | 3.02 | 2.24 | 2.63 | 0.33 |  | 0.00077 |
| 6 | F-43 | M | 60 | | Left Forearm | MVK:c.935A>G | NNS-gDNA | 1.21 | 1.13 |  |  | 1.17 | 0.05 |  |  |
|  |  |  |  | |  |  | LT-gDNA | 1.28 | 1.23 |  |  | 1.25 | 0.03 | 0.18347 |  |
|  |  |  |  | |  |  | NNS-cDNA | 1.26 | 1.17 | 1.51 | 1.18 | 1.28 | 0.16 |  |  |
|  |  |  |  | |  |  | LT-cDNA | 6.51 | 6.87 | 6.72 | 6.29 | 6.60 | 0.26 |  | 0.00001 |
|  |  |  |  | | Left Thigh | MVK:c.935A>G | NNS-gDNA | 1.02 | 1.12 |  |  | 1.07 | 0.07 |  |  |
|  |  |  |  | |  |  | LT-gDNA | 1.59 | 1.67 |  |  | 1.63 | 0.06 | 0.01302 |  |
|  |  |  |  | |  |  | NNS-cDNA | 1.92 | 1.92 | 1.84 | 1.74 | 1.85 | 0.09 |  |  |
|  |  |  |  | |  |  | LT-cDNA | 6.31 | 7.80 | 7.79 | 7.40 | 7.33 | 0.70 |  | 0.00050 |
| 7 | F-38 | M | 68 | | Left Forearm | MVK:c.1093T>A | NNS-gDNA | 0.96 | 0.97 |  |  | 0.97 | 0.01 |  |  |
|  |  |  |  | |  |  | LT-gDNA | 1.00 | 1.04 |  |  | 1.02 | 0.03 | 0.16218 |  |
|  |  |  |  | |  |  | NNS-cDNA | 0.74 | 0.72 | 0.93 | 0.67 | 0.76 | 0.11 |  |  |
|  |  |  |  | |  |  | LT-cDNA | 0.82 | 0.80 | 0.95 | 0.79 | 0.84 | 0.08 |  | 0.13194 |
| 8 | F-35 | F | 29 | | Right Forearm | MVD: c.746T>C | NNS-gDNA | 1.41 | 1.13 |  |  | 1.27 | 0.20 |  |  |
|  |  |  |  | |  |  | LT-gDNA | 0.87 | 0.49 |  |  | 0.68 | 0.27 | 0.13166 |  |
|  |  |  |  | |  |  | NNS-cDNA | 0.62 | 0.65 | 1.49 | 0.94 | 0.92 | 0.41 |  |  |
|  |  |  |  | |  |  | LT-cDNA | 0.71 | 0.74 | 0.57 | 0.93 | 0.74 | 0.15 |  | 0.40471 |
| 9 | S-28 | F | 38 | | Right Leg | MVD: c.746T>C | NNS-gDNA | 1.09 | 0.76 |  |  | 0.92 | 0.23 |  |  |
|  |  |  |  | |  |  | LT-gDNA | 0.99 | 1.47 |  |  | 1.23 | 0.34 | 0.40279 |  |
|  |  |  |  | |  |  | NNS-cDNA | 0.87 | 0.92 | 0.93 | 0.79 | 0.88 | 0.04 |  |  |
|  |  |  |  | |  |  | LT-cDNA | 2.61 | 3.26 | 2.57 | 3.24 | 2.92 | 0.46 |  | 0.00004 |
| 10 | F-33 | M | 45 | | Left Forearm | MVD:c.875A>G | NNS-gDNA | 1.23 | 1.24 |  |  | 1.24 | 0.01 |  |  |
|  |  |  |  | |  |  | LT-gDNA | 1.14 | 1.10 |  |  | 1.12 | 0.02 | 0.02546 |  |
|  |  |  |  | |  |  | NNS-cDNA | 0.88 | 1.00 | 1.19 | 0.96 | 1.01 | 0.13 |  |  |
|  |  |  |  | |  |  | LT-cDNA | 1.51 | 1.72 | 1.62 | 1.62 | 1.62 | 0.08 |  | 0.00070 |
| 11 | F-37 | M | 62 | | Trunk | MVD:c.875A>G | NNS-gDNA | 1.10 | 1.07 |  |  | 1.09 | 0.02 |  |  |
|  |  |  |  | |  |  | LT-gDNA | 1.39 | 1.26 |  |  | 1.33 | 0.09 | 0.06534 |  |
|  |  |  |  | |  |  | NNS-cDNA | 1.01 | 1.20 | 0.82 | 0.78 | 0.95 | 0.19 |  |  |
|  |  |  |  | |  |  | LT-cDNA | 2.97 | 2.15 | 2.68 | 2.97 | 2.69 | 0.39 |  | 0.00591 |
| 12 | S-36 | F | 56 | | Left Forearm | FDPS:c.684+1G>A | NNS-gDNA | 1.06 | 1.00 |  |  | 1.03 | 0.04 |  |  |
|  |  |  |  | |  |  | LT-gDNA | 1.08 | 1.04 |  |  | 1.06 | 0.03 | 0.50496 |  |
|  |  |  |  | |  |  | NNS-cDNA | 0.06 | 0.04 | 0.05 | 0.06 | 0.05 | 0.01 |  |  |
|  |  |  |  | |  |  | LT-cDNA | 0.10 | 0.11 | 0.11 | 0.13 | 0.11 | 0.01 |  | 0.00376 |
|  |  |  |  | |  |  |  |  |  |  |  |  |  |  |  |

Notes:

1. A1 and A2 measurements designate two PCR repeats for genomic DNA sample or the first reverse transcript cDNA product; B1 and B2 measurements designate two PCR repeats for the second reverse transcript cDNA product.
2. p value1 and p value2 indicates the significance level for comparison of the mutant/wild allelic ratios between NNS-gDNA and LT-gDNA and between NNS-cDNA and LT-cDNA from a pairwise tissue set respectively.
3. NNS, neighboring normal-appearing skin; LT, lesion tissue; gDNA, genomic DNA; cDNA, complementary DNA ; AEI, allelic expression imbalance; M/W, mutant/wild; STD, standard deviation.
